# Memristic Characteristics from Bistable to Tristable Memory with Controllable Charge Trap Carbon Nanotubes

**DOI:** 10.3390/nano8020114

**Published:** 2018-02-17

**Authors:** Lei Li, Dianzhong Wen

**Affiliations:** Key Laboratories of Senior-Education for Electronic Engineering, Heilongjiang University, Harbin 150080, China

**Keywords:** tristable memory, memristic characteristics, multi-bit, fluorescence spectrum

## Abstract

The incorporation of the one-dimensional carbon nanomaterial carbon nanotubes (CNTs) in poly(methyl methacrylate) (PMMA) was found to successfully develop a resistive switching. It implements memristic characteristics which shift from bistable to tristable memory. The localized current pathways in the organic nanocomposite layers for each intermediate resistive state (IRS) are attributed to the trapping mechanism consistent with the fluorescent measurements. Multi-bit organic memories have attracted considerable interest, which provide an effective way to increase the memory density per unit cell area. This study will be useful for the development and tuning of multi-bit storable organic nanocomposite memory device systems.

## 1. Introduction

Intelligent functional materials designed for organic memory devices, fueled by information explosion, must cater to ultra-high-density data storage [1,2]. In fact, optical and magnetic storage technologies based on inorganic semiconductors traditionally suffer from a variety of restrictions, including an incremental demand for memory materials together with downscaling of the cell size. The advent of resistive random-access memories (RRAMs), as one of the most prominent transformations, has played a critical role in favor of structure simplicity, layer-by-layer stacking, low power consumption, and capability for the storage of multi-conductive states [3,4]. Multi-bit organic memories have attracted considerable interest for their high data storage capacity. In spite of advances, the search for multi-bit memory materials still remains a formidable challenge. The majority of research on organic memory devices has been concentrated on binary memory with bistably electronic organic materials. Tremendous efforts have been made to develop a variety of memory materials and boost the memory performance of devices such as a high OFF/ON ratio, long-term retention, and switching speed. The capability for multi-bit information storage provides an effective way to increase the memory density per unit cell area.

In light of electrical bistability, existing organic memory devices primarily accommodate in a binary system with the data storage capacity limited to 2^n^. Exceeding two conductive states in memory devices can be considered as one of the most effective methods to boost the data storage [5,6,7,8,9,10,11,12,13,14]. Several organic memory devices have recently been reported to fulfill ternary memory behavior under an external electric field, which facilitates a powerful increase in data-storage density. Multi-bit memories dependent on small molecules with a D-A structure are found in organometallics with two or more reversible oxidation states. Nevertheless, memories based on polymer offer several benefits, including solution processing and higher structural diversity. In contrast with binary memories, multi-bit memories are much more challenging, as suitable materials are not accessible and the underlying mechanisms are controversial. Many mechanisms have been applied to explain the performance of organic storage behaviors, including metal-filament formation, field-induced charge transfer, molecular redox, conformational change, and charge trapping. It has been theorized that ternary storage materials containing different trap depths may be a precondition for storage [15,16,17,18,19,20]. Various traps have a critical role in the formation of conducting paths in the organic active layers by comparing current–voltage characteristics with well-established charge transport mechanisms, such as Poole–Frenkel conduction, space charge limited current, and Fowler–Nordheim tunneling.

In this study, for further investigation, the changes of the memristic characteristics with different contents of carbon nanotubes (CNTs) were observed in the composite of PMMA and CNTs. For resistive memory systems established by nanocomposite materials, organic/inorganic nanoclusters embedded in organic matrices can be perceived as the charge trapping elements. More specifically, this paper aims to observe the electronic dynamics, thereby enabling a better understanding of memristic characteristics from bistable to tristable memory in organic nanocomposite memory systems.

## 2. Materials and Methods 

The COOH-functionalized multiwalled carbon nanotubes (MWCNT-COOH) were purchased from Hengqiu Tech. Inc., Suzhou, China, the carboxylic content of which reached 2 wt %. The nanocomposites of PMMA (purchased from ARKEMA, Colombes, France) and MWCNT-COOH were spin-coated on indium tin oxide (ITO)-coated glass substrates to manufacture resistive switching memory devices. Firstly, the ITO-coated glass substrates whose conductivity ranged from 6 Ω/sq to 9 Ω/sq were ultrasonicated for 1 h, and were successively cleaned in acetone, methanol, and ethanol solvents. The insulating polymer, PMMA, was dissolved in a chloroform (10 mg/mL) solution. Different weight % of MWCNT-COOH were added to the PMMA solution. After that, the mixed solution of PMMA and MWCNT-COOH was ultrasonicated for 2 h, then spun on ITO substrates at a speed of 3000 rpm for 60 s. Next, the hybrid films consisting of PMMA and MWCNT-COOH were prepared on ITO-coated glass substrates in ambient air. To evaporate solvents, the films were kept at 60 °C for 24 h in vacuum. Subsequently, a 300-nm-thick Ni layer was deposited on top of the thin film at room temperature (300 K) by thermal evaporation technique with a shadow mask. The evaporation was performed at 1 × 10^−5^ Torr of pressure.

An FEI Sirion (Philips, Holland, The Netherlands) field emission scanning electron microscope (FESEM) was used to characterize the cross-section of the hybrid films with MWCNT-COOH dispersed in polymer matrix. A NanoMap 500LS 3D Profilometer (aep Technology, Santa Clara, CA, USA) was adopted to measure the thickness of the hybrid films. Micrographs of MWCNT-COOH embedded in polymer were acquired using a JEM-2100 (JOEL, Tokyo, Japan) transmission electron microscopy (TEM). Raman spectroscopy (LabRam HR800 Raman Spectrometer; Horiba Jobin Yvon, Villeneuve-d’Ascq, France) was coupled with an Olympus BX41 microscope fitted with 50× objective, employing a 514.5 nm laser source. The spectral resolution was 0.35 cm^−1^, while the spatial resolution was 1 μm and 2 μm, respectively, from horizontal and vertical angles. The spectral peaks were fitted by Gaussian model with the program Origin 8.0. Thermogravimetric analysis (TGA; TA Instruments, New Castle, DE, USA) and differential scanning calorimetry (DSC; TA Instruments, USA) were used to analyze the thermal properties of MWCNT-COOH. TGA was implemented under a nitrogen atmosphere at heating rates of 10 °C/min, 15 °C/min, and 20 °C/min. An AVENTES fiber optic spectrometer (AvaSpec-HS1024×58TEC-USB2 and AvaSpec-NIR256-1.7TEC; Avantes, Holland, The Netherlands) was used to record. The electrical characteristics of the MWCNT-COOH-based resistive switching were measured by a semiconductor parameter analyzer (Keithley 4200SCS; Keithley, Solon, OH, USA) at ambient temperature. During electrical measurements of the memory devices, the Ni top layer was grounded, while an electrical bias was applied to the ITO bottom electrodes. All measurements of the devices were conducted under ambient conditions, without any encapsulation.

## 3. Results and Discussion

### 3.1. TGA, DSC, and Raman Measurement

**Figure 1:**
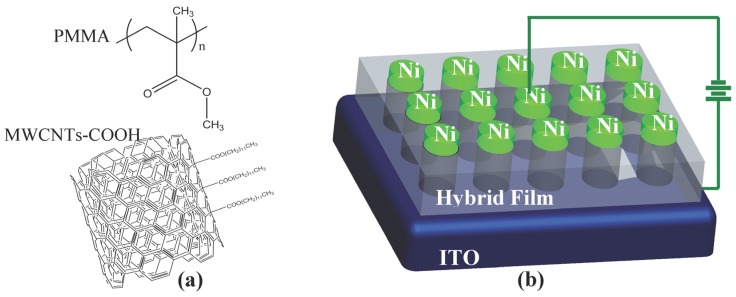
Resistive switching of indium tin oxide (ITO)/PMMA:MWCNT-COOH/Ni. (**a**) The structure of PMMA and a schematic representation of MWCNT-COOH; (**b**) Configuration of the sandwiched resistive switching ITO/PMMA:MWCNT-COOH/Ni. MWCNT-COOH: COOH-functionalised multiwalled carbon nanotube; PMMA: poly(methyl methacrylate).

**Figure 2:**
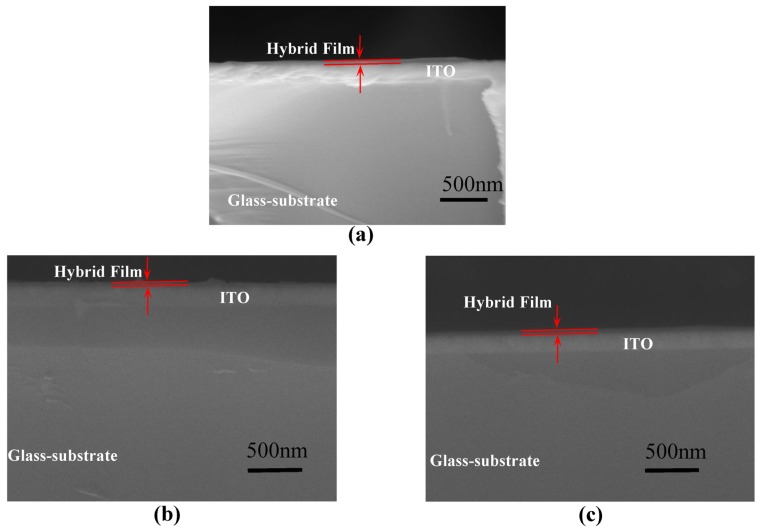
Cross-sectional field emission scanning electron microscope (FESEM) images of PMMA:MWCNT-COOH nanocomposite films with the distinct contents of MWCNT-COOH (**a**) 1%; (**b**) 2%; and (**c**) 3%, respectively.

**Figure 3:**
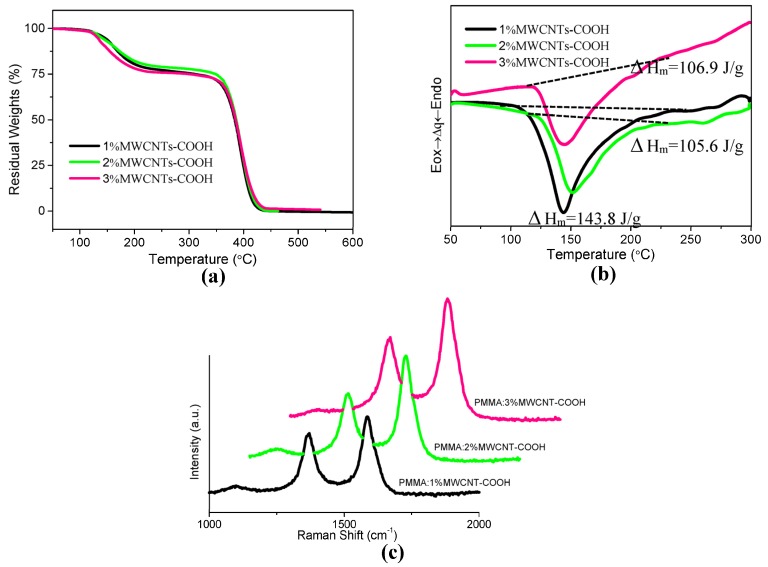
(**a**) Thermogravimetric analysis (TGA) curves and (**b**) Differential scanning calorimetry (DSC) curves at a heating rate of 10 ℃·min^−1^, measured in a nitrogen atmosphere, for the nanocomposites with the mass weight 1%, 2%, and 3% of MWCNT-COOH; (**c**) Raman spectra of the hybrid films with the different mass weight of MWCNT-COOH.

The structure of PMMA and a schematic representation of MWCNT-COOH are indicated in Figure 1a. The molecular structure of PMMA is a linear polymeric chain of methyl methacrylate monomers, which act as the constitutional unit. For MWCNTs, the combination between adjacent carbon atoms is in the form of the C-C covalent bond, which is one of the strongest chemical bonds. The ITO/PMMA: MWCNT-COOH/Ni sandwiched structure is shown in Figure 1b. The cross-section of the nanocomposite film with 1%, 2%, and 3% MWCNT-COOH content was characterized by FESEM, and the corresponding micrographs are shown in Figure 2. A profilometer was used to measure the thickness of all the films, which was estimated to be 30 nm. Figure 3a shows TGA curves of the corresponding PMMA: MWCNT-COOH nanocomposites. Based on this figure, all of them exhibited a good thermal stability with a weight loss of about 5% at the decomposition temperatures of 145 °C (PMMA: 1%MWCNT-COOH), 140 °C (PMMA: 2%MWCNT-COOH), and 132 °C (PMMA: 3%MWCNT-COOH). It exhibited a two-step degradation: the first step was due to the loss of remaining water molecules and oxygenating functional groups. The second degradation step (370–450 °C) involved the pyrolysis of the remaining oxygen-containing groups as well as the burning of the ring carbon [21]. It can be seen that the nanocomposites completely degraded at ~400 °C. DSC measurements of the nanocomposites were performed, and the results obtained are shown in Figure 3b. The figure illustrates the corresponding glass-transition process at *T*_g_ = 121.0 °C, 130.1 °C, and 122.7 °C, the corresponding melting temperatures of *T*_m_ = 143.6 °C, 151.3 °C, and 145.4 °C, and the corresponding melting enthalpies Δ*H*_m_ = 143.8 J/g, 105.6 J/g, and 106.9 J/g, respectively. Raman spectrum measurements of the as-prepared hybrid films with different mass weights of MWCNT-COOH were performed, as shown in Figure 3c. The Raman spectra with specific peaks for MWCNTs include a D-band and a G-band. A shift of the D peak of MWCNTs arose separately at 1364 cm^−^^1^, 1366 cm^−^^1^, and 1370 cm^−^^1^ as the incremental content of MWCNT-COOH, while that of the G peak happened separately at 1578 cm^−^^1^, 1582 cm^−^^1^, and 1586 cm^−^^1^. The shift may be related to the increased concentration of CNTs. For all of the nanocomposite films with 1%, 2%, and 3% MWCNT-COOH, the corresponding intensity ratio of the D and G bands *I*_D_/*I*_G_ was calculated to be 0.86.

### 3.2. Current–Voltage (I–V) Characteristics of the Memory Devices

**Figure 4:**
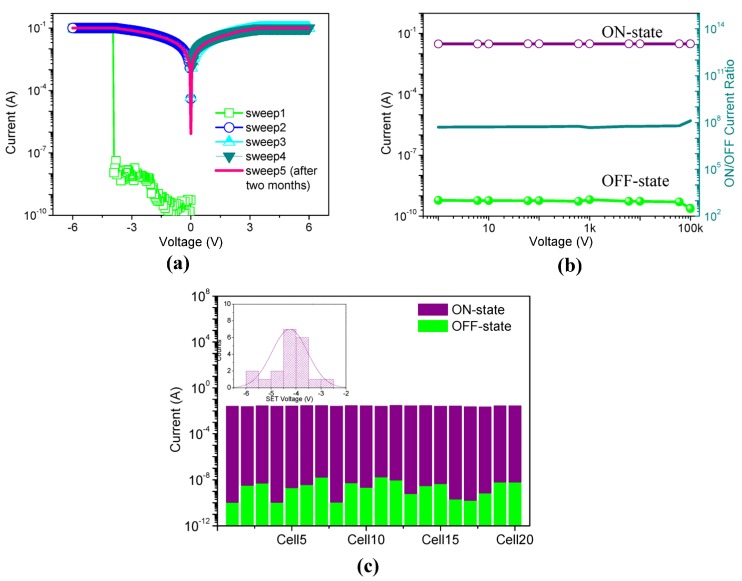
Memristic characteristics of ITO/PMMA:MWCNT-COOH/Ni with the content of MWCNT-COOH 1%. (**a**) *I*–*V* characteristics; (**b**) Retention characteristics for ON and OFF states under a constant stress of −1 V; (**c**) Device-to-Device distribution of *I*_ON_ and *I*_OFF_, and *V*_th_ in Inset.

In this work, we have also investigated how MWCNT-COOH content would impact on the memory performance of ITO/PMMA:MWCNT-COOH/Ni. The memristic characteristics are illustrated in Figure 4.

A typical *I*–*V* curve of the resistive switching with 1% MWCNT-COOH is demonstrated in Figure 4a. During the applied voltage sweep, the measured current values were plotted in a logarithmic scale, with the compliance current kept in check at 10^−1^ A. It implements a binary write-once read-many-times (WORM) memory behavior with an ON/OFF current ratio greater than 10^7^. At first, the resistive switching was in a high resistance state (HRS or “0” state) with current levels of 10^−10^–10^−9^ A. The current surged at the threshold voltage *V*_th_ = −3.9 V, switching from OFF-state to ON-state. Then, the device retained a low resistance state (LRS or “1” state), whether applying a reverse voltage sweep or even removing the applied electrical field. The transition between HRS (OFF-state or “0” state) and LRS (ON-state or “1” state) was switchable and bistable. Then, the sample was kept in the lab atmosphere where the humidity and temperature of the test could be maintained in the same condition. After two months (sweep 5), the same cell was measured again, sweeping from −6 V to 6 V, which remained in the ON-state. The retention characteristics of the ON and OFF states showed that the ON state was stable beyond 10^5^ s under a constant stress of −1 V (Figure 4b). We measured twenty cells of ITO/PMMA:MWCNT-COOH/Ni to evaluate the repeatability of *I*–*V* characteristics. At a voltage of −1 V, Figure 4c shows the device-to-device profile of *I*_ON_ and *I*_OFF_ without considerable deviations. The primary devices exhibited binary electrical transition signals with no ternary performance observed. In the inset, *V*_th_ of the devices was mostly located from −2.9 V to −5.7 V. The reproducibility of MWCNT-COOH-based cells is feasible for the reliable resistive switching with high ON/OFF ratio. Owing to the insulating nature of pure PMMA, the hybrid film with a doping level of MWCNT-COOH below 1% performed as an insulator.

**Figure 5:**
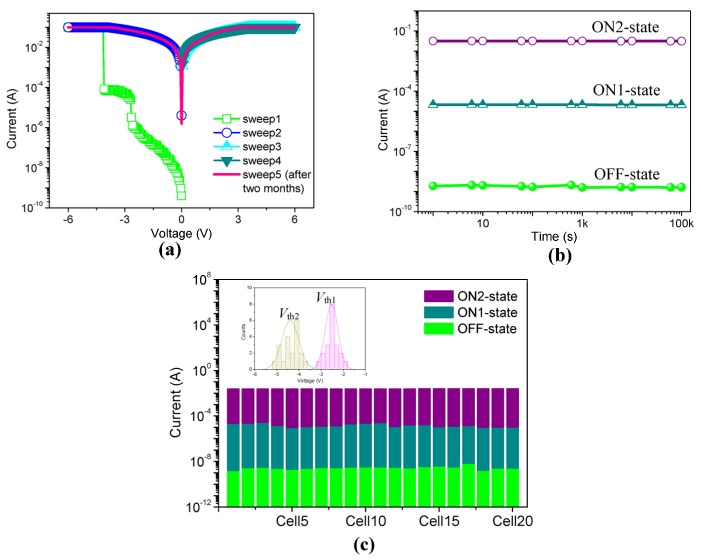
Memristic characteristics of ITO/PMMA:MWCNT-COOH/Ni with the content of MWCNT-COOH 2%. (**a**) *I*–*V* characteristics; (**b**) Retention characteristics for ON and OFF states under a constant stress of −1 V; (**c**) Device-to-Device distribution of *I*_ON_ and *I*_OFF_, and *V*_th1_ and *V*_th2_ in the inset.

**Figure 6:**
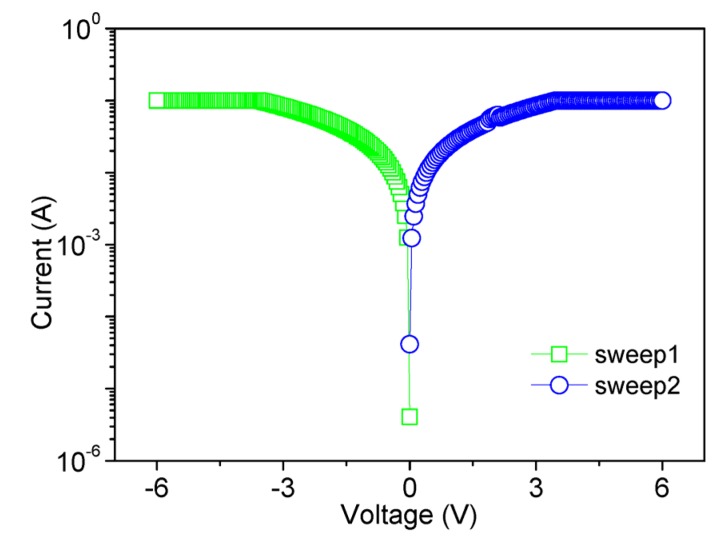
*I*–*V* characteristics of ITO/PMMA:3%MWCNT-COOH/Ni with the content of MWCNT-COOH.

Compared with the 1% MWCNT-COOH film, the memristic characteristics of the hybrid film with 2% of MWCNT-COOH presented tristable memory behavior, shown in Figure 5. As for its *I*–*V* characteristics, the device initially exhibited HRS (OFF-state or “0” state). When a negative voltage (0 to −6 V, sweep 1) was applied to the ITO electrode, two abrupt current increases were observed at switching threshold voltages of *V*_th1_ = −2.6 V and *V*_th2_ = −4.2 V, indicating that the device went through disrupt electrical transitions from OFF-state to an intermediate-conductive state (ON1-state or “1” state), and then to LRS (ON2-state or “2” state). The cell remained LRS during a subsequent scan from 0 to −6 V (sweep 2), even after the power was turned off or by applying a reverse bias (sweep 3 and sweep 4). The result suggests that the resistive switching with content of MWCNT-COOH 2% can be employed as ternary WORM device. The current ratios of three distinct conductive states were roughly 1:10^3^:10^7^, identical with “0”, “1”, and “2” states. All three states presented good stability under a stress test of −1 V for the retention time beyond 10^5^ s, exhibited in Figure 5b. No significant degradation in current for “0”, “1”, and “2” states was seen during the test. Besides the individual cell performance, the reproductivity of *I*–*V* behaviors has been considered as well. *I*_ON2_, *I*_ON1_, and *I*_OFF_ at *V* = −1 V (for each device) are depicted in Figure 5c. For twenty independent cells, *V*_th1_ was found to be in the range from −1.9 V to −3.1 V, and *V*_th2_ was between −3.8 V and −5.1 V, showing reasonable separations between the two switching threshold voltages, as plotted in Inset. The distinguishable two threshold voltages, long retention time, and good reproducibility of three conductivity states demonstrate that it is a promising candidate active material for ternary resistive memory devices. At ambient temperature, the electrical measurement of the multi-bit resistive switching was carried out in air. With a further increase in the content of MWCNT-COOH (3%), it showed only a single high-conductivity state without switching phenomenon (Figure 6). This indicates that the device had no data storage ability. In summary, the electrical properties of PMMA:MWCNT-COOH devices varied from bistable to tristable memory behavior and further to conductor behavior with increasing MWCNT content.

### 3.3. Proposed Memory Mechanisms

**Figure 7:**
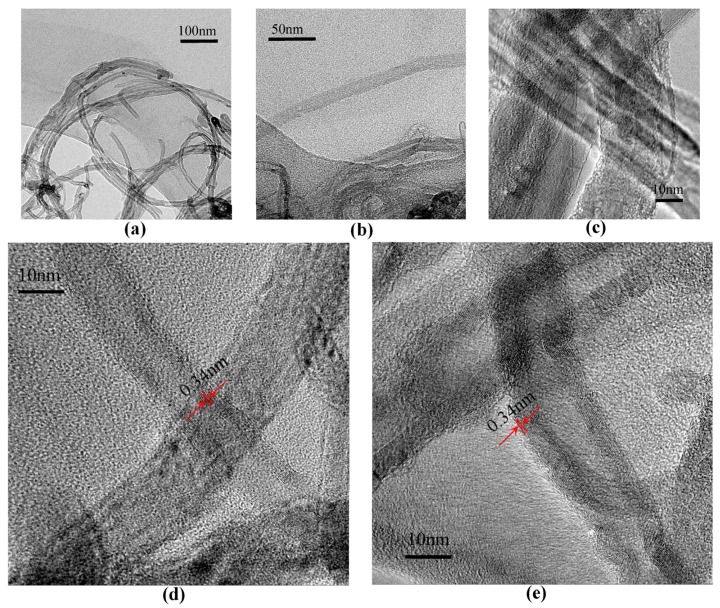
TEM images of PMMA and MWCNT-COOH. (**a**–**c**) TEM patterns of pristine MWCNT-COOH with different ranges of vision; (**d**,**e**) TEM patterns of PMMA:MWCNT-COOH blends at different positions of the same TEM grid.

**Figure 8:**
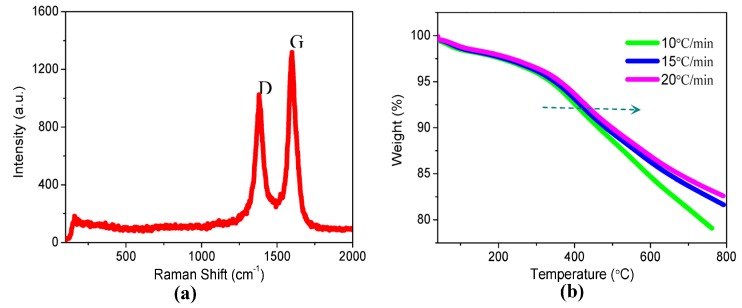
Characterization of MWCNT-COOH. (**a**) Raman spectrum of MWCNT-COOH; (**b**) TGA curves of MWCNT-COOH with different rising rates of temperature.

The one-dimensional carbon nanomaterial along its axis, CNTs, mostly affect the electrical behavior of the hybrid films because of the insulator-like PMMA. To gain insight into memory behaviors from bistable to tristable states, TEM micrographs of pristine MWCNT-COOH are shown in Figure 7a–c, in which the samples of MWCNT-COOH were ultrasonicated in ethanol. These figures demonstrate that pristine MWCNT-COOH possesses several micrometers of length and several nanometers of diameter. It is comprised of multilayers of single-walled CNTs in the coaxial shell. The hybrid films were manufactured by spin-coating the nanocomposite solution with 2% MWCNT-COOH on TEM grids. Figure 7d,e are TEM images of PMMA and MWCNT-COOH blend at different positions of the same TEM grid. The layer-to-layer distance approached the interlayer spacing of graphene 0.34 nm. It was observed that the number of multilayers ranged from 10 to 20. Spin-casting of the nanocomposites on ITO substrates resulted in a thin hybrid film with planar and random orientation of CNTs. The Raman spectrum of MWCNT-COOH possesses two characteristic bands in Figure 8a. The G peak at 1598.4 cm^−1^ is ascribed to *E*_2g_ mode of vibration in the graphite planar, whose strength reflects the degree of graphitization. In addition, the D peak at 1380.3 cm^−1^ is associated with the defects and the disorder plane of MWCNT-COOH. The intensity ratio of the D and G bands *I*_D_/*I*_G_ is an essential factor to evaluate the quality of CNTs. The *I*_D_/*I*_G_ ratio was estimated to be 0.78, which indicates that the tubes were crystalline in nature [22]. Different from CNTs, the ratio *I*_D_/*I*_G_ for the hybrid films in Figure 3c may be influenced by the PMMA component. With the range of rising temperature from 40 °C to 800 °C, TGA curves of MWCNT-COOH in Figure 8b illustrate that MWCNT-COOH were relatively active at heating rates of 10 °C/min, 15 °C/min and 20 °C/min. It was found that the samples of MWCNT-COOH exhibited good thermal stability, with the 5%-mass-loss temperature at 338 °C, 348 °C, and 362 °C, respectively. The carboxylic group successively decomposed when the weight loss continued until 800 °C. This process stems from the oxidation reaction of MWCNT-COOH, which was not completely oxidized, since the weight loss of MWCNT-COOH was in the range of 18–21% at 800 °C.

**Figure 9:**
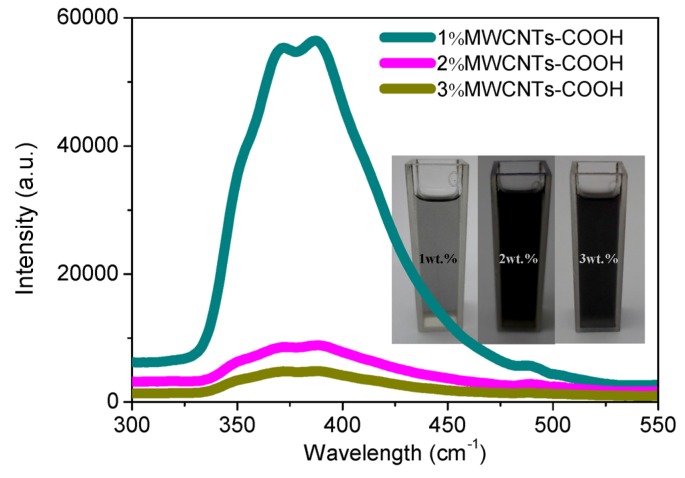
Fluorescence spectra of PMMA:MWCNT-COOH blends in solutions with different mass fractions of MWCNT-COOH. Inset: photographs of the homogeneous solutions.

In order to investigate the interaction between PMMA and MWCNT-COOH, optical fiber spectroscopic experiments were conducted, scanning from 300–550 nm, and the results obtained are shown in Figure 9. In these experiments, the optical fiber of the spectroscope was placed perpendicular to that of the light source. For the homogeneous solution of PMMA:MWCNT-COOH blends (10 mg/mL), Figure 9 demonstrates the fluorescence spectra associated with the mass fraction of MWCNT-COOH 1%, 2%, and 3%. After varying the content of MWCNTs, the quenching phenomena were observed from the fluorescence spectra with the optical excitation wavelength 390 nm. MWCNT-COOH hardly leads to the conformation change of PMMA, with the fact that neither red shift nor blue shift of the maximal excited wavelength was observed in various mass fractions of MWCNT-COOH. A number of fluorescent polymers exhibit high sensitivity to CNTs quenchers, anticipated to quench fluorescence by electron or energy transfer [23,24,25,26,27,28]. When the quenching effect occurs at a “trap site” where the quencher MWCNT-COOH associates with the polymer repeat units, the quenching effect of PMMA:MWCNT-COOH blends appears in conjunction with the strong interaction between PMMA and MWCNT-COOH with energy migration and/or delocalization within MWCNT-COOH.

Apart from TEM, Raman spectrum and TGA, the fluorescent quenching occurred when PMMA noncovalently attached to the surface of CNTs. The resistive switching mechanism is based on MWCNT-COOH, and therefore can be closely linked with traps under a bias voltage. These traps have distinct energy levels that can be sequentially filled by increasing voltage, which contributes to tristable resistive switching behavior. The device behavior is associated with charge carrier trapping and inter-CNT hopping by virtue of the effective distance between neighboring CNTs or the CNT content in the hybrid film [29]. With the CNT content increasing from 1% to 3%, the distance between isolated nanotubes was significantly decreased. The diameter of individual CNTs is suitable for charge hopping between individual CNTs. Due to the strong electron-withdrawing ability of CNTs, a great deal of electron pathways can be formed throughout the entire nanocomposite. A larger number of carrier pathways are responsible for the significant increment in the ON-state current and the consequent growth in *I*_ON_/*I*_OFF_ once the threshold voltage was reached. A further increase in the CNT content up to 2% led to a simultaneous decrease in distance between isolated nanotubes. Charge carrier transport along the electron pathways by inter-CNT hopping becomes easier and occurs earlier (intermediate state) than that in the binary WORM device, leading to less charge carriers being trapped prior to switching. Even though the applied voltage was removed, the charge largely remained trapped since the deep trapping level prevented the detrapping process. ITO/PMMA:3%MWCNT-COOH/Ni had a higher concentration of MWCNT-COOH, which made it easier to form pathways for charge hopping, initially resulting in the ON-state.

## 4. Conclusions

This work demonstrated a MWCNT-COOH-based resistive switching translating from bistable into tristable behaviors through controllable charge trap CNTs. The one-dimensional carbon nanomaterial along its axis, CNTs, mostly affected the electrical behavior of the hybrid films. The resistive switching mechanism based on MWCNT-COOH can be closely associated with traps under a bias voltage with the fact that the fluorescent quenching occurs. These results may offer a new strategy to heighten the performance of materials and stability of electronic devices by introducing CNTs-based component in organic molecules for organic multi-bit data storage devices.
